# Characterizing the relationship between coronavirus disease 2019 (COVID-19) and central-line–associated bloodstream infection (CLABSI) and assessing the impact of a nursing-focused CLABSI reduction intervention during the COVID-19 pandemic

**DOI:** 10.1017/ice.2022.203

**Published:** 2023-07

**Authors:** Michael A. Ben-Aderet, Meghan S. Madhusudhan, Pishoy Haroun, Matthew J.P. Almario, Ryan Raypon, Sharon Fawcett, Julie Johnson, Anita Girard, Todd Griner, Lorraine Sheffield, Jonathan D. Grein

**Affiliations:** 1 Department of Hospital Epidemiology, Cedars-Sinai Medical Center, Los Angeles, California; 2 University of California Los Angeles, Los Angeles, California; 3 Department of Nursing, Cedars-Sinai Medical Center, Los Angeles, California

## Abstract

**Objective:**

To examine the impact of SARS-CoV-2 infection on CLABSI rate and characterize the patients who developed a CLABSI. We also examined the impact of a CLABSI-reduction quality-improvement project in patients with and without COVID-19.

**Design:**

Retrospective cohort analysis.

**Setting:**

Academic 889-bed tertiary-care teaching hospital in urban Los Angeles.

**Patients or participants:**

Inpatients 18 years and older with CLABSI as defined by the National Healthcare Safety Network (NHSN).

**Intervention(s):**

CLABSI rate and patient characteristics were analyzed for 2 cohorts during the pandemic era (March 2020–August 2021): COVID-19 CLABSI patients and non–COVID-19 CLABSI patients, based on diagnosis of COVID-19 during admission. Secondary analyses were non–COVID-19 CLABSI rate versus a historical control period (2019), ICU CLABSI rate in COVID-19 versus non–COVID-19 patients, and CLABSI rates before and after a quality- improvement initiative.

**Results:**

The rate of COVID-19 CLABSI was significantly higher than non–COVID-19 CLABSI. We did not detect a difference between the non–COVID-19 CLABSI rate and the historical control. COVID-19 CLABSIs occurred predominantly in the ICU, and the ICU COVID-19 CLABSI rate was significantly higher than the ICU non–COVID-19 CLABSI rate. A hospital-wide quality-improvement initiative reduced the rate of non–COVID-19 CLABSI but not COVID-19 CLABSI.

**Conclusions:**

Patients hospitalized for COVID-19 have a significantly higher CLABSI rate, particularly in the ICU setting. Reasons for this increase are likely multifactorial, including both patient-specific and process-related issues. Focused quality-improvement efforts were effective in reducing CLABSI rates in non–COVID-19 patients but were less effective in COVID-19 patients.

Central-line–associated bloodstream infection (CLABSI) is a common hospital-acquired infection that contributes to patient morbidity, mortality, increased patient length of stay, and hospital cost.^1,2^ CLABSIs are reported by hospitals to the National Healthcare Safety Network (NHSN) using standardized criteria that allows for benchmarking.^1^ The body of literature directed toward CLABSI prevention is robust; public health and professional societies have published guidance for clinicians and healthcare facilities that include such evidence-based practices as implementation of sterile insertion bundles, chlorhexidine (CHG) bathing, maintenance bundles and guidance for sterile line access.^3^ Between 2015 and 2020, there had been a nationwide trend toward reduction in CLABSI in US hospitals, and in 2019 the NHSN recorded a 31% decline in standardized infection ratio (SIR) from the 2015 baseline.^4^

The onset of the COVID-19 pandemic marked an end to this positive trend, and a subsequent increase in the incidence of CLABSI (as well as other HAI) has been widely documented in different settings.^4–7^ Nationwide, the CLABSI SIR as documented by the NHSN increased significantly, beginning in the second quarter of 2020, reversing the steady reduction in prior years.^2^ At our institution we experienced a similar trend in CLABSI: between 2019 and 2020, our CLABSI rate more than doubled. Although studies have correlated increased incidence of CLABSI during the pandemic with increases in COVID-19 patient census and hospital size,^6,7^ few data have been published regarding the patient-level risk of CLABSI in patients with COVID-19.

We performed a retrospective cohort analysis to examine the impact of infection with SARS-CoV-2 on CLABSI rate at our hospital, as well as the characteristics of patients admitted with a diagnosis of COVID-19 who developed a CLABSI. We also examined the impact of an institutional quality-improvement initiative aimed at reducing these infections.

## Methods

The analysis was performed at an 889-bed, tertiary-care, teaching hospital in urban Los Angeles. Prior to the pandemic, baseline CLABSI rates had been consistently below national benchmarks, and robust CLABSI prevention efforts included the use of sterile insertion bundles, alcohol-impregnated port protectors, universal daily CHG bathing, as well as real-time case assessments for all CLABSI. Throughout the analysis, CLABSI surveillance was performed by the department of hospital epidemiology. All positive blood cultures were reviewed by full-time nurse infection preventionists employed by the department who had been trained in NHSN surveillance methodology and criteria.

All CLABSIs were included in the analysis if the infection met NHSN criteria.^1^ CLABSIs excluded from analysis were those infections in patients aged <18 years and those involving extracorporeal membrane oxygen (ECMO CLABSI), ventricular-assist device (VAD CLABSI), or mucosal barrier injury (MBI CLABSI) as defined by the NHSN.^1^

In our analysis, we designated March 1, 2020, through August 31, 2021, as the pandemic period, given that March 2020 was the onset of known community spread of COVID-19 in Los Angeles. We compared the data from this period to those from the full calendar year immediately preceding the pandemic, January 1, 2019–December 31, 2019, designated as our prepandemic period. A complete calendar year was chosen to represent the normal spectrum of CLABSI seen at the institution in the prepandemic period. We use the term “COVID-19 CLABSIs” to refer to CLABSIs that occurred during an admission with a molecular test positive for severe acute respiratory coronavirus virus 2 (SARS-CoV-2) or a physician flag for active COVID-19 illness. CLABSIs that occurred in these patients after removal of COVID-19 transmission-based precautions were considered COVID-19 CLABSIs for the purposes of this analysis. “Non–COVID-19 CLABSIs” refer to CLABSIs in all other patients during the pandemic period. We define “ICU CLABSI” as a CLABSI that occurred in any ICU, using NHSN attribution criteria.^1^

In our primary analysis, we compared COVID-19 and non–COVID-19 CLABSI rates (infections per 1,000 catheter days) and patient characteristics for all admitted patients. To control for any baseline changes to our non–COVID-19 CLABSI population, we performed a secondary comparison of non–COVID-19 CLABSIs with all CLABSIs in the prepandemic period. Due to the predominance of ICU CLABSIs in the COVID-19 CLABSI cohort, we also performed secondary analyses for ICU CLABSIs. We compared COVID-19 ICU CLABSIs with non–COVID-19 ICU CLABSI and non–COVID-19 ICU CLABSIs with all ICU CLABSIs in the prepandemic period. Due to very small numbers, we did not perform a subanalysis of non-ICU COVID-19 CLABSIs. Lastly, we compared all COVID-19 and non–COVID-19 CLABSIs during the pandemic period before and after the implementation of a CLABSI-reduction quality-improvement initiative.

Designated COVID-19 cohort units were used for the care of all patients with COVID-19 throughout the study periods. The cohort ICU is in the medical ICU, and care is provided by medical ICU nurses and physicians, including house staff. From January 2020 through February 2021 however, large numbers of COVID-19 ICU patients necessitated the expansion of the COVID-19 ICU into 3 other ICU areas. Use and duration of transmission-based precautions for COVID-19 followed CDC guidance; prior to September 29, 2020, test-based criteria were used to determine duration of isolation, and a non–test-based criteria were used thereafter.^8^

The quality-improvement work was a hospital-wide project initiated in September 2020 by the department of nursing and hospital epidemiology focused on CLABSI reduction in all patients. This project consisted of resuming real-time case assessments for all CLABSIs, a long-standing practice that was placed on hold in March–August 2020 due to redirection of infection control efforts to COVID-19–related work. In addition to the resumption of this work, the intervention also involved novel interventions: monthly distribution of CLABSI-specific incidence and process-measure dashboards to the leadership of all nursing units as well as targeted weekly line rounds on the COVID-19 ICU by a “line champions” team. This team consisted of bedside nurses selected as champions, a designated infection preventionist, and a nursing unit manager. Rounds consisted of charting and bedside audits with immediate feedback and education to the bedside nurse that focused on catheter maintenance, including dressing integrity, use of alcohol-impregnated port protectors, timely removal of peripheral catheters, tubing changes and labeling, and proper techniques. These elements were chosen as the area of focus due to a perceived drift in practice during the early months of the pandemic. Rounding results were communicated to hospital leadership and the entire nursing staff of the COVID-10 ICU via a weekly e-mail.

All SARS-CoV-2 testing was done via nucleic acid amplification. Data sources included NSHN CLABSI surveillance data and patient-level comorbidity data identified through coding using criteria for the Charlson comorbidities.^9^ CLABSI rates were compared using mid-*P* exact test based on Poisson distribution. Patient characteristics were compared using χ^2^ tests for categorical variables, the mid-*P* exact test based on hypergeometric distribution for device utilization, and an independent sample *t* test or Wilcoxon-Mann-Whitney test for continuous variables. Subjective data collected by infection preventionists for each CLABSI during real-time case reviews with clinical staff were also reviewed. This study was performed for quality improvement purposes and was not considered human-subject research.

## Results

### Primary analysis

In the pandemic period, the COVID-19 CLABSI rate was 4.75 per 1,000 catheter days, compared with a non–COVID-19 CLABSI rate of 0.63 (relative risk of CLABSI, 7.5; *P* < .0001). COVID-19 CLABSI patients were older, had higher rates of diabetes, were more likely to have been admitted to an ICU during their admission (98% vs 54%; *P* < .0001), were more likely to have been in an ICU at the time of the CLABSI diagnosis (89% vs 29%; *P* < .0001), and had a higher in-house mortality rate (49% vs 21%; *P* = .0025) (Table 1).

**Table 1.** COVID-19 CLABSI Versus Non–COVID-19 CLABSI in the Pandemic Period, March 2020–August 2021

Variable	Non–COVID-19 CLABSI (N=52), No. (%)^a^	COVID-19 CLABSI (N=55), No. (%)^a^	*P* Value
CLABSI rate per 1,000 central-line days (n/N)	0.63 (52/82,686)	4.75 (55/11,581)	**<.0001**
**Patient demographics**			
Age, average y	53	64	**.0016**
Sex, female	19 (37)	21 (38)	.8606
**Race**			
Asian	7 (13)	3 (5)	
Black/African-American	13 (25)	7 (13)	
Other	8 (15)	12 (22)	
White	24 (46)	33 (60)	
**Charlson comorbidities**			
Myocardial infarction history	12 (23)	18 (33)	.2667
Congestive heart failure	19 (37)	26 (47)	.2609
Peripheral vascular disease	11 (21)	6 (11)	.1474
Cerebrovascular disease	10 (19)	7 (13)	.3577
Dementia	4 (8)	2 (4)	.3621
Chronic pulmonary disease	11 (21)	18 (33)	.1783
Rheumatic disease	1 (2)	4 (7)	.1900
Peptic ulcer disease	3 (6)	3 (5)	.9436
Mild liver disease	9 (17)	13 (24)	.4182
Diabetes without complication	13 (25)	34 (62)	**.0001**
Diabetes with complication	13 (25)	24 (44)	**.0428**
Hemiplegia	6 (12)	6 (11)	.9179
Renal disease	26 (50)	32 (58)	.3959
Malignancy (leukemia and lymphoma)	12 (23)	5 (9)	.0479
Moderate or severe liver disease	5 (10)	1 (2)	.0797
Metastatic solid tumor	4 (8)	4 (7)	.9343
AIDS/HIV	1 (2)	0 (0)	.3015
Charlson comorbidity score, median	5	5	.6587
**Encounter**			
Hospital LOS, median d	38	40	.4374
ICU during encounter	28 (54)	54 (98)	**<.0001**
ICU at the time of CLABSI	15 (29)	49 (89)	**<.0001**
Time to CLABSI, median d	18	16	.5577
In-house mortality	11 (21)	27 (49)	**.0025**
**Line details**			
Central line duration during the encounter, median d	26	22	.4967
Device utilization ratio, catheter days/patient days	0.57	0.66	**<.0001**
Dialysis line attributed to CLABSI	21 (40)	19 (35)	.5327
Femoral line attributed to CLABSI	12 (23)	3 (5)	**.0087**
PICC line attributed to CLABSI	14 (27)	5 (9)	**.0158**
Port line attributed to CLABSI	7 (13)	1 (2)	**.0221**
PA catheter line attributed to CLABSI	3 (6)	0 (0)	.0708
Arterial line present on infection date	14 (27)	49 (89)	**<.0001**
**Organism**			
Gram-positive organisms	28 (54)	29 (53)	.9077
Methicillin-resistant *S. aureus*	1 (2)	1 (2)	.9681
Methicillin-susceptible *S. aureus*	4 (8)	0 (0)	**.0360**
Coagulase-negative *Staphylococcus*	12 (23)	13 (24)	.9455
* Enterococcus* spp	9 (17)	14 (25)	.3052
Gram-negative organisms	14 (27)	6 (11)	**.0337**
* Candida* spp	12 (23)	25 (45)	**.0150**
Polymicrobial CLABSI	4 (8)	9 (16)	.1700

Note. ICU, intensive care unit; CLABSI, central-line–associated bloodstream infection; LOS, length of stay; AIDS, acquired immune deficiency syndrome; HIV, human immunodeficiency virus; PICC, peripherally inserted central catheter; pulmonary artery.

a Units unless otherwise specified.

Regarding central-line type, COVID-19 CLABSI patients were less likely to have femoral catheters or long-term lines, such as peripherally inserted central catheter (PICC) or port, attributed to the CLABSI. Arterial line utilization at the time of the CLABSI infection diagnosis was more frequent in COVID-19 CLABSI patients (89% vs 27%; *P* < .0001). The central-line utilization ratio was higher in the COVID-19 CLABSI cohort (0.66 vs 0.57; *P* < .0001), but we did not detect a significant difference in median time to CLABSI from line insertion.

*Candida* spp were more prevalent in COVID-19 CLABSIs (45% vs 23%; *P* = .0150), whereas gram-negative organisms were more prevalent in non–COVID-19 CLABSIs (27% vs 11%; *P* = .0337). We did not detect a significant difference in rates of CLABSI with coagulase-negative *Staphylococcus*, *Enterococcus*, methicillin-resistant *S. aureus*, or polymicrobial CLABSI, but patients with COVID-19 CLABSI had a lower rate of methicillin-susceptible *Staphylococcus aureus*.

### Secondary analyses

*Non–COVID-19 CLABSI versus prepandemic CLABSI.* We found no significant difference between the prepandemic CLABSI rate and the non–COVID-19 CLABSI rate (0.62 vs 0.63; *P* = .9655) (Supplementary Table 1). Compared to the prepandemic period, the patients in the pandemic period with non–COVID-19 CLABSI were younger (63 vs 53 years; *P* = .0092), were more likely to have femoral catheters (0% vs 23%; *P* = .0013) and had lower catheter utilization (0.73 vs 0.57 catheter/patient days; *P* < .0001). We found no significant differences Charlson comorbidity index scores, median central-line duration, time to CLABSI, or organism categories.

### COVID-19 ICU CLABSI and non–COVID-19 ICU CLABSI

In the pandemic period, the COVID-19 ICU CLABSI rate was 6.31, compared to a non–COVID-19 ICU CLABSI rate of 0.60 (relative risk of CLABSI, 10.5; *P* < .0001) (Table 2). We detected no difference in age, sex, hospital length of stay, ICU length of stay, time to CLABSI, or mortality. Patients with ICU COVID-19 CLABSI were more likely to have diabetes and less likely to have moderate-to-severe liver disease. Unlike the larger cohort, the organism breakdown and use of long-term catheters were similar between the ICU CLABSI cohorts, though the non–COVID-19 ICU CLABSI cohort had a higher rate of femoral catheter and pulmonary-artery catheter use. Arterial line at the time of the CLABSI diagnosis was again more frequent in COVID-19 CLABSI patients (96% vs 53%; *P* < .0001), as was the central-line utilization ratio (0.65 vs 0.45; *P* < .0001).

**Table 2.** COVID-19 ICU CLABSI Versus Non–COVID-19 ICU CLABSI in the Pandemic Period, March 2020–August 2021

Variable	Non–COVID-19 ICU CLABSIs (n=15), No. (%)^a^	COVID-19 ICU CLABSIs (n=49), No. (%)^a^	*P* Value
ICU CLABSI rate per 1,000 central-line days (n/N)	0.60 (15/24,866)	6.31 (49/7,763)	**<.0001**
**Patient demographics**			
Age, average y	52	64	.0708
Sex, female	5 (33)	20 (41)	.6032
**Race**			
Asian	1 (7)	2 (4)	
Black/African-American	7 (47)	7 (14)	
Other	1 (7)	11 (22)	
White	6 (40)	29 (59)	
**Charlson comorbidities**			
Myocardial infarction history	4 (27)	15 (31)	.7698
Congestive heart failure	9 (60)	23 (47)	.3760
Peripheral vascular disease	4 (27)	4 (8)	.0580
Cerebrovascular disease	3 (20)	6 (12)	.4497
Dementia	0 (0)	2 (4)	.4266
Chronic pulmonary disease	3 (20)	14 (29)	.5107
Rheumatic disease	0 (0)	3 (6)	.3263
Peptic ulcer disease	2 (13)	2 (4)	.1952
Mild liver disease	3 (20)	11 (22)	.8409
Diabetes without complication	4 (27)	30 (61)	**.0189**
Diabetes with complication	4 (27)	20 (41)	.3219
Hemiplegia	2 (13)	6 (12)	.9112
Renal disease	10 (67)	28 (57)	.5111
Malignancy (leukemia and lymphoma)	2 (13)	5 (10)	.7340
Moderate or severe liver disease	3 (20)	1 (2)	**.0119**
Metastatic solid tumor	0 (0)	4 (8)	.2531
AIDS/HIV	0 (0)	0 (0)	1.0000
Charlson comorbidity score, median	6	5	.8296
**Encounter**			
Hospital LOS, median d	31	39	.6142
ICU LOS, median d	25	25	1.0000
ICU during encounter	15 (100)	49 (100)	1.0000
ICU at the time of CLABSI	15 (100)	49 (100)	1.0000
Time to CLABSI, median d	17	15	.3030
In-house mortality	5 (33)	24 (49)	.2868
**Line variables**			
Central-line duration during the encounter, median, d	26	21	.3209
Device utilization ratio, catheter days/patient days	0.45	0.65	**<.0001**
Dialysis line attributed to CLABSI	9 (60)	17 (35)	.0808
Femoral line attributed to CLABSI	5 (33)	3 (6)	**.0053**
PICC line attributed to CLABSI	3 (20)	4 (8)	.1987
Port line attributed to CLABSI	0 (0)	0 (0)	1.0000
PA catheter line attributed to CLABSI	2 (13)	0 (0)	**.0094**
Arterial line present on infection date	8 (53)	47 (96)	**<.0001**
**Organism**			
Gram-positive organisms	6 (40)	25 (51)	.4549
Methicillin-resistant *S. aureus*	0 (0)	1 (2)	.5771
Methicillin-susceptible *S. aureus*	0 (0)	0 (0)	1.0000
Coagulase-negative *Staphylococcus*	2 (13)	10 (20)	.5390
* Enterococcus* spp	4 (27)	13 (27)	.9917
Gram-negative organisms	4 (27)	6 (12)	.1783
* Candida* spp	5 (33)	23 (47)	.3527
Polymicrobial CLABSI	0 (0)	9 (18)	.0734

Note. ICU, intensive care unit; CLABSI, central-line–associated bloodstream infection; LOS, length of stay; AIDS, acquired immune deficiency syndrome; HIV, human immunodeficiency virus.

a Units unless otherwise specified.

#### Non–COVID-19 ICU CLABSI and prepandemic ICU CLABSI

We found no significant difference between the prepandemic ICU CLABSI rate and the non–COVID-19 ICU CLABSI rate (0.9 vs 0.6; *P* = .2586) (Supplementary Table 2). The patients with ICU CLABSI in the prepandemic period were older (68 vs 52 years; *P* = .0346) and more likely to have dementia (24% vs 0%; *P* = .0446), whereas pandemic period non–COVID-19 ICU CLABSIs were more likely attributed to femoral catheters (0% vs 33%; *P* = .0096). We found no significant differences in CLABSI rates, median central-line duration, or organisms observed (gram-positive, gram-negative, or *Candida*).

#### COVID-19 and non–COVID-19 CLABSIs before and after the quality-improvement intervention

**Fig. 1.**
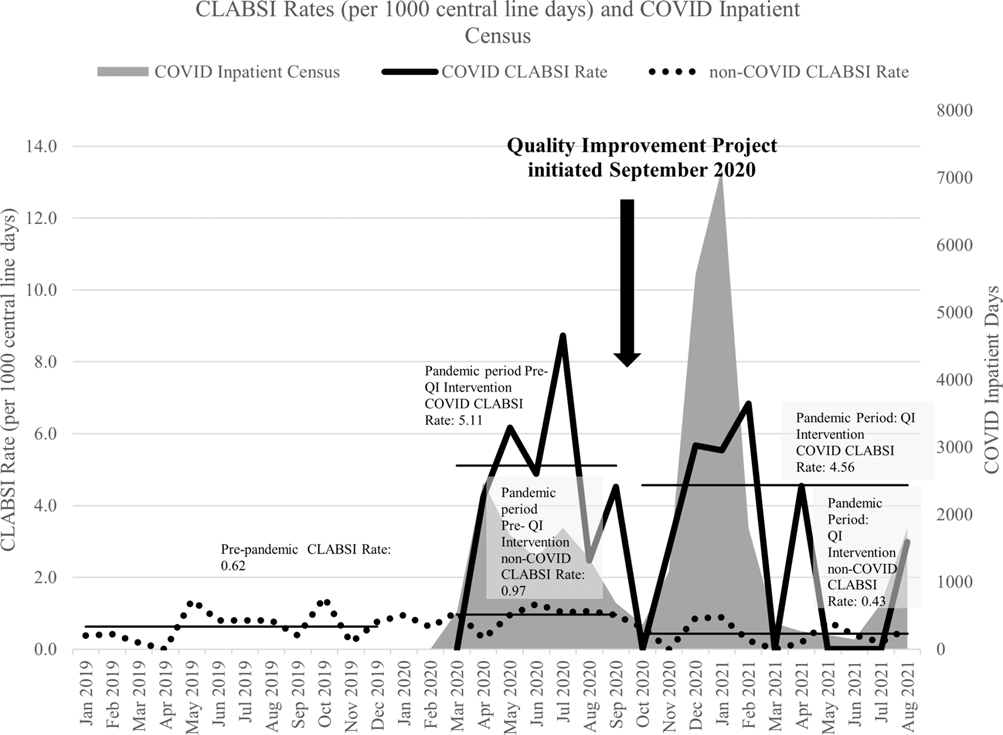
Comparing monthly COVID-19 and non–COVID-19 CLABSI rates with inpatient COVID-19 census. COVID-19 CLABSI rates trended higher during and immediately after months of high COVID-19 inpatient census both before and after implementation of a quality improvement project.

After implementation of the quality-improvement intervention, the COVID-19 CLABSI rate did not show a significant decrease from 5.11 to 4.56 (*P* = .6804), whereas the non–COVID-19 CLABSI rate decreased significantly from 0.97 to 0.43 (*P* = .0034) (Fig. 1). We did not detect a significant difference between the prepandemic CLABSI rate and the non–COVID-19 CLABSI rate before the intervention. In both the pre- and postintervention periods, the COVID-19 CLABSI rate increased during and immediately after months of higher COVID-19 census. Variation in the non–COVID-19 CLABSI rate did not appear to be correlated with COVID-19 census.

Comparing patient characteristics before and after the quality-improvement initiative, we found no relevant statistically significant differences (Supplementary Table 3).

## Discussion

In this patient-level analysis of patients with CLABSI with and without COVID-19, we observed a significantly higher CLABSI rate in patients with COVID-19, particularly in the ICU. Compared to historical controls, the CLABSI rate did not increase in non–COVID-19 patients, which suggests that the increase in CLABSI risk is likely related to factors associated with their COVID-19 diagnosis. A focused, hospital-wide quality improvement initiative significantly reduced CLABSI rates in non–COVID-19 patients, but the impact on CLABSI rates in COVID-19 patients was minimal. Additionally, COVID-19 CLABSI rates appeared to increase during times of high COVID-19 census both before and after the intervention.

Our analysis showed patient-level differences in the COVID-19 and non–COVID-19 CLABSIs that may partially explain this trend. Compared to the non–COVID-19 CLABSI cohort, COVID-19 CLABSI patients had more comorbidities and markers of critical illness: they were older, were more likely to be diabetic, were more likely to have any ICU stay, were more likely to use an arterial line use, and were far more likely to be in the ICU at the time of CLABSI.

To isolate the effect of critical illness, we performed the secondary analysis of pandemic period COVID-19 versus non–COVID-19 ICU CLABSIs, and surprisingly, we detected an even higher rate of COVID-19 CLABSI and a higher relative risk. Additionally, when comparing the patient-level characteristics between the ICU CLABSI cohorts, most of the differences observed in the primary analysis resolved, including line type, organism distribution, ICU length of stay, hospital length of stay, and mortality. Quite clearly, critical illness and ICU status alone do not fully explain the high COVID-19 ICU CLABSI rate.

A finding that remained in the analysis of ICU CLABSI was the significant difference in arterial catheter usage: 96% of COVID-CLABSI in the ICU occurred while an arterial catheter was in place. At our hospital, arterial catheters are routinely placed when pronation therapy is used, which underlies the use in this cohort. Arterial catheters are likely an underrepresented source of hospital-onset BSI,^11^ so the significant increase in usage in the COVID-19 population may have contributed to the increased CLABSI rates. It is also possible that other COVID-19 ICU–specific novel COVID-19 treatments, such as pronation and/or immune modulation with steroids and/or tocilizumab, may have contributed to CLABSI respectively through mechanical disruption of dressings and increased susceptivity to bacterial infection.

In addition to patient characteristics, process-related factors likely contributed to increased COVID-19 CLABSI rates. Increased PPE burden and decreased time in the room may have led to gaps in normal CLABSI prevention practices in the COVID-19 ICU, which was a designated area. Other novel practices with unclear consequences include pronation therapy and the placement of IV pumps outside the rooms of COVID-19 ICU patients, which was done frequently at our institution and may have affected the maintenance of clean and protected IV access. Potentially compounding these factors, the COVID-19 ICU suffered serious staffing challenges as the rapid rise and fall in numbers of COVID-19 cases in discrete surges increased the burden of caring for these patients, requiring coordinated large numbers of cohort ICU beds in addition to trained ICU nurses and physicians. The higher central-line utilization ratio in the COVID-19 CLABSI population illustrates that, despite the similar comorbidities noted in Table 2, there was an increased care burden in this population. These concerns are reinforced by the associated of increased COVID-19 CLABSI rate during and immediately after months of high COVID-19 census.

The quality-improvement intervention was primarily focused on data feedback to all areas and targeted line rounds in the COVID-19 ICU, focused on maintenance. Although we achieved significant reductions in non–COVID-19 CLABSI, we detected no significant effect on the rate of COVID-19 CLABSI, the target of the line rounds. Whereas Moss et al^11^ demonstrated success in reducing COVID-19 CLABSI in an ICU over 2 months using audits and maintenance bundles, our experience was fundamentally different due to the number of patients in our COVID-19 ICU, the size of our institution, and the breadth of our postintervention follow-up across 11 months and 2 distinct infection surges. Although the improvement observed in non–COVID-19 CLABSI rates reflects the impact of interventions on the general patient population, the minimal improvement in COVID-19 CLABSI, despite the focused rounds, is concerning. Possible explanations include unique issues with this population not addressed by the intervention, including some that may have been exacerbated by times of high COVID-19 census, particularly the surge in November 2020–February 2021, when almost all of these CLABSIs occurred.

There has been speculation that a redirection of infection prevention work to focus on COVID-19 during the pandemic led to the national increases in CLABSI.^12^ Our data do not support this hypothesis. We were able to maintain stable and low CLABSI rates in the non–COVID-19 population throughout the pandemic, and the COVID-19 CLABSI rate was persistently high even after redirection of infection control and nursing resources to CLABSI prevention with the quality-improvement intervention.

The analysis has several notable strengths. This study was conducted at a large hospital with pre-existing robust CLABSI prevention efforts and low baseline CLABSI rates. We were the first hospital in Los Angeles to admit a patient with COVID-19, with large numbers of admissions and high acuity throughout the pandemic, allowing a large sample size of COVID-19 CLABSI for a single center. Numerous analyses of the increase in CLABSI have been conducted since the start of the pandemic, but this is the first to examine both the patient-level impact of the diagnosis of COVID-19 on CLABSI rates, as well as the effect of a targeted reduction intervention. These findings will lay the groundwork for further analysis of COVID-19 CLABSI causality and explorations of prevention strategies.

This study had several limitations. Study design, by necessity, was a nonexperimental retrospective review of events that unfolded during a pandemic; thus, the environment was not controlled. SARS-CoV-2 testing, therapeutics, healthcare worker perceptions of risk, and some infection control practices changed over time, especially early in the pandemic response. Another limitation was the lack of objective data on bedside catheter insertion or maintenance practice during the early months of the pandemic because audits were deferred due to redirection of infection control resources.

Our data demonstrate that patients hospitalized for COVID-19 are at significantly higher risk of developing a CLABSI than other patients, particularly in the ICU setting. Reasons for this increased risk are likely multifactorial, including both patient-specific and process-related issues. Focused quality improvement efforts were effective in reducing CLABSI rates in non–COVID-19 patients, but they were less effective in COVID-19 patients. More research is needed to better characterize the relationship between the COVID-19 census and COVID-19 CLABSI, to better understand the specific risk factors associated with CLABSI in COVID-19 patients, and to determine which measures may be more effective in reducing this risk.

## Supplementary material

For supplementary material accompanying this paper visit https://doi.org/10.1017/ice.2022.203. click here to view supplementary material
