# Supplementary material for: Characterizing the relationship between coronavirus disease 2019 (COVID-19) and central-line–associated bloodstream infection (CLABSI) and assessing the impact of a nursing-focused CLABSI reduction intervention during the COVID-19 pandemic
Source: Infect Control Hosp Epidemiol. 2022 Aug 31;44(7):1108–15. doi: 10.1017/ice.2022.203 (PMC10369209; doi:10.1017/ice.2022.203)
Supplement: Supplementary file 1 [file S0899823X22002033sup001.docx]

Supplementary Table 1: Pre-pandemic CLABSI vs. Non-COVID CLABSI

|  |  | **2019  All CLABSIs n=39** | **Mar 2020 - Aug 2021 Non-COVID CLABSI n=52** | **p-value** |
| --- | --- | --- | --- | --- |
| CLABSI Rate | CLABSI Rate (per 1000 central line days) | 0.62  (39/62653) | 0.63  (52/82686) | 0.9655 |
| Patient Demographics | Age (average, years) | 63 | 53 | **0.0092** |
|  | Female | 36% (n=14) | 37% (n=19) | 0.9498 |
|  | Race | Asian - 13% (n=5) Black/African-American - 15% (n=6) Other - 15% (n=6) White - 56% (n=22) | Asian - 13% (n=7) Black/African-American - 25% (n=13) Other - 15% (n=8) White - 46% (n=24) |  |
| Charlson Comorbidities | Myocardial Infarction history | 23% (n=9) | 23% (n=12) | 1.0000 |
|  | Congestive Heart Failure | 49% (n=19) | 37% (n=19) | 0.2437 |
|  | Peripheral Vascular Disease | 13% (n=5) | 21% (n=11) | 0.3014 |
|  | Cerebrovascular Disease | 18% (n=7) | 19% (n=10) | 0.8766 |
|  | Dementia | 10% (n=4) | 8% (n=4) | 0.6690 |
|  | Chronic Pulmonary Disease | 10% (n=4) | 21% (n=11) | 0.1656 |
|  | Rheumatic Disease | 0% (n=0) | 2% (n=1) | 0.3839 |
|  | Peptic Ulcer Disease | 5% (n=2) | 6% (n=3) | 0.8944 |
|  | Mild Liver Disease | 13% (n=5) | 17% (n=9) | 0.5571 |
|  | Diabetes without complication | 41% (n=16) | 25% (n=13) | 0.1045 |
|  | Diabetes with complication | 36% (n=14) | 25% (n=13) | 0.2601 |
|  | Hemiplegia | 8% (n=3) | 12% (n=6) | 0.5430 |
|  | Renal Disease | 56% (n=22) | 50% (n=26) | 0.5444 |
|  | Malignancy (Leukemia and lymphoma) | 33% (n=13) | 23% (n=12) | 0.2781 |
|  | Moderate or Severe Liver Disease | 13% (n=5) | 10% (n=5) | 0.6285 |
|  | Metastatic Solid Tumor | 21% (n=8) | 8% (n=4) | 0.0737 |
|  | AIDS/HIV | 0% (n=0) | 2% (n=1) | 0.3839 |
|  | Charlson Comorbidity Score (median) | 7 | 5 | 0.0845 |
| Encounter | Hospital LOS (median, days) | 30 | 38 | 0.5570 |
|  | ICU during encounter | 69% (n=27) | 54% (n=28) | 0.1375 |
|  | ICU at the time of CLABSI | 44% (n=17) | 29% (n=15) | 0.1449 |
|  | Time to CLABSI (median, days) | 16 | 18 | 0.6302 |
|  | In-house mortality | 38% (n=15) | 21% (n=11) | 0.0705 |
| Line details | Central line duration during the encounter (median, days) | 21 | 26 | 0.3604 |
|  | Device Utilization Ratio (catheter days/patient days) | 0.73 | 0.57 | **<0.0001** |
|  | Dialysis line attributed to CLABSI | 26% (n=10) | 40% (n=21) | 0.1419 |
|  | Femoral line attributed to CLABSI | 0% (n=0) | 23% (n=12) | **0.0013** |
|  | PICC line attributed to CLABSI | 46% (n=18) | 27% (n=14) | 0.0573 |
|  | Port line attributed to CLABSI | 10% (n=4) | 13% (n=7) | 0.6425 |
|  | PA catheter line attributed to CLABSI | 10% (n=4) | 6% (n=3) | 0.4266 |
|  | Arterial line present on infection date | 23% (n=9) | 27% (n=14) | 0.6761 |
| Organism | Gram-Positive organisms | 62% (n=24) | 54% (n=28) | 0.4631 |
|  | Methicillin resistant *Staphylococcus aureus* | 5% (n=2) | 2% (n=1) | 0.3968 |
|  | Methicillin susceptible *Staphylococcus aureus* | 15% (n=6) | 8% (n=4) | 0.2456 |
|  | Coagulase-negative *Staphylococcus* | 33% (n=13) | 23% (n=12) | 0.2781 |
|  | Enterococcus spp | 10% (n=4) | 17% (n=9) | 0.3415 |
|  | Gram-Negative organisms | 15% (n=6) | 27% (n=14) | 0.1884 |
|  | Candida spp | 23% (n=9) | 23% (n=12) | 1.0000 |
|  | Polymicrobial CLABSI | 5% (n=2) | 8% (n=4) | 0.6257 |

Supplementary Table 2: Pre-Pandemic ICU-CLABSI vs. Non-COVID ICU CLABSI

|  |  | **2019  ICU CLABSIs n=17** | **Mar 2020 - Aug 2021 Non-COVID ICU CLABSIs n=15** | **p-value** |
| --- | --- | --- | --- | --- |
| CLABSI Rate | ICU CLABSI Rate (per 1000 central line days) | 0.90  (17/18811) | 0.60 (15/24866) | 0.2586 |
| Patient Demographics | Age (average, years) | 68 | 52 | **0.0346** |
|  | Female | 24% (n=4) | 33% (n=5) | 0.5382 |
|  | Race | Asian - 12% (n=2) Black/African-American - 12% (n=2) Other - 12% (n=2) White - 65% (n=11) | Asian - 7% (n=1) Black/African-American - 47% (n=7) Other - 7% (n=1) White - 40% (n=6) |  |
| Charlson Comorbidities | Myocardial Infarction history | 35% (n=6) | 27% (n=4) | 0.5993 |
|  | Congestive Heart Failure | 71% (n=12) | 60% (n=9) | 0.5291 |
|  | Peripheral Vascular Disease | 24% (n=4) | 27% (n=4) | 0.8379 |
|  | Cerebrovascular Disease | 29% (n=5) | 20% (n=3) | 0.5395 |
|  | Dementia | 24% (n=4) | 0% (n=0) | **0.0446** |
|  | Chronic Pulmonary Disease | 18% (n=3) | 20% (n=3) | 0.8649 |
|  | Rheumatic Disease | 0% (n=0) | 0% (n=0) | 0.3263 |
|  | Peptic Ulcer Disease | 12% (n=2) | 13% (n=2) | 0.8935 |
|  | Mild Liver Disease | 12% (n=2) | 20% (n=3) | 0.5220 |
|  | Diabetes without complication | 59% (n=10) | 27% (n=4) | 0.0673 |
|  | Diabetes with complication | 47% (n=8) | 27% (n=4) | 0.2344 |
|  | Hemiplegia | 6% (n=1) | 13% (n=2) | 0.4705 |
|  | Renal Disease | 65% (n=11) | 67% (n=10) | 0.9072 |
|  | Malignancy (Leukemia and lymphoma) | 6% (n=1) | 13% (n=2) | 0.4705 |
|  | Moderate or Severe Liver Disease | 18% (n=3) | 20% (n=3) | 0.8649 |
|  | Metastatic Solid Tumor | 0% (n=0) | 0% (n=0) | 0.2531 |
|  | AIDS/HIV | 0% (n=0) | 0% (n=0) | 0.3293 |
|  | Charlson Comorbidity Score (median) | 7 | 6 | 0.3149 |
| Encounter | Hospital LOS (median, days) | 37 | 31 | 0.5647 |
|  | ICU LOS (median, days) | 27 | 25 | 0.7481 |
|  | ICU during encounter | 100% (n=17) | 100% (n=15) | 1.0000 |
|  | ICU at the time of CLABSI | 100% (n=17) | 100% (n=15) | 1.0000 |
|  | Time to CLABSI (median, days) | 18 | 17 | 0.5961 |
|  | In-house mortality | 53% (n=9) | 33% (n=5) | 0.2645 |
| Line details | Central line duration during the encounter (median, days) | 29 | 26 | 0.7769 |
|  | Device Utilization Ratio (catheter days/patient days) | 0.74 | 0.45 | **<0.0001** |
|  | Dialysis line attributed to CLABSI | 41% (n=7) | 60% (n=9) | 0.2879 |
|  | Femoral line attributed to CLABSI | 0% (n=0) | 33% (n=5) | **0.0096** |
|  | PICC line attributed to CLABSI | 24% (n=4) | 20% (n=3) | 0.8096 |
|  | Port line attributed to CLABSI | 0% (n=0) | 0% (n=0) | 1.0000 |
|  | PA catheter line attributed to CLABSI | 18% (n=3) | 13% (n=2) | 0.7373 |
|  | Arterial line present on infection date | 53% (n=9) | 53% (n=8) | 0.9823 |
| Organism | Gram-Positive organisms | 53% (n=9) | 40% (n=6) | 0.4641 |
|  | Methicillin resistant *Staphylococcus aureus* | 12% (n=2) | 0% (n=0) | 0.1701 |
|  | Methicillin susceptible *Staphylococcus aureus* | 6% (n=1) | 0% (n=0) | 0.3399 |
|  | Coagulase-negative *Staphylococcus* | 35% (n=6) | 13% (n=2) | 0.1522 |
|  | Enterococcus spp | 12% (n=2) | 27% (n=4) | 0.2811 |
|  | Gram-Negative organisms | 24% (n=4) | 27% (n=4) | 0.8379 |
|  | Candida spp | 24% (n=4) | 33% (n=5) | 0.5382 |
|  | Polymicrobial CLABSI | 6% (n=1) | 0% (n=0) | 0.3399 |

Supplementary Table 3: COVID and non-COVID CLABSI rates and characteristics before and after implementation of a quality improvement intervention.

|  |  | **Pre-Intervention Mar 2020-Sep 2020 COVID CLABSIs n=20** | **Intervention Oct 2020-Aug 2021 COVID CLABSIs n=35** | **p-value** | **Pre-Intervention Mar 2020-Sep2020 Non-COVID CLABSIs n=30** | **Intervention Oct 2020-Aug2021 Non-COVID CLABSIs n=22** | **p-value** |
| --- | --- | --- | --- | --- | --- | --- | --- |
| CLABSI Rate | CLABSI Rate (per 1000 central line days) | 5.11 (20/3913) | 4.56 (35/7668) | 0.6804 | 0.97 (30/31004) | 0.43 (22/51682) | **0.0034** |
| Patient Demographics | Age (average, years) | 65 | 62 | 0.4042 | 53 | 53 | 0.9469 |
|  | Female | 35% (n=7) | 40% (n=14) | 0.7135 | 23% (n=7) | 55% (n=12) | **0.0209** |
|  | Race | Asian - 0% (n=0) Black/African-American - 15% (n=3) Other - 20% (n=4) White - 65% (n=13) | Asian - 9% (n=3) Black/African-American - 11% (n=4) Other - 23% (n=8) White - 57% (n=20) |  | Asian - 13% (n=4) Black/African-American - 17% (n=5) Other - 13% (n=4) White - 57% (n=17) | Asian - 14% (n=3) Black/African-American - 36% (n=8) Other - 18% (n=4) White - 32% (n=7) |  |
| Charlson Comorbidities | Myocardial Infarction history | 40% (n=8) | 29% (n=10) | 0.3849 | 23% (n=7) | 23% (n=5) | 0.9591 |
|  | Congestive Heart Failure | 65% (n=13) | 37% (n=13) | **0.0465** | 33% (n=10) | 41% (n=9) | 0.5751 |
|  | Peripheral Vascular Disease | 0% (n=0) | 17% (n=6) | **0.0498** | 23% (n=7) | 18% (n=4) | 0.6532 |
|  | Cerebrovascular Disease | 15% (n=3) | 11% (n=4) | 0.7022 | 17% (n=5) | 23% (n=5) | 0.5838 |
|  | Dementia | 0% (n=0) | 6% (n=2) | 0.2761 | 7% (n=2) | 9% (n=2) | 0.7459 |
|  | Chronic Pulmonary Disease | 35% (n=7) | 31% (n=11) | 0.7860 | 17% (n=5) | 27% (n=6) | 0.3549 |
|  | Rheumatic Disease | 5% (n=1) | 9% (n=3) | 0.6237 | 3% (n=1) | 0% (n=0) | 0.3872 |
|  | Peptic Ulcer Disease | 5% (n=1) | 6% (n=2) | 0.9107 | 10% (n=3) | 0% (n=0) | 0.1265 |
|  | Mild Liver Disease | 15% (n=3) | 29% (n=10) | 0.2544 | 23% (n=7) | 9% (n=2) | 0.1798 |
|  | Diabetes without complication | 55% (n=11) | 66% (n=23) | 0.4314 | 20% (n=6) | 32% (n=7) | 0.3309 |
|  | Diabetes with complication | 40% (n=8) | 46% (n=16) | 0.6810 | 27% (n=8) | 23% (n=5) | 0.7459 |
|  | Hemiplegia | 20% (n=4) | 6% (n=2) | 0.1021 | 13% (n=4) | 9% (n=2) | 0.6362 |
|  | Renal Disease | 55% (n=11) | 60% (n=21) | 0.7176 | 50% (n=15) | 50% (n=11) | 1.0000 |
|  | Malignancy (Leukemia and lymphoma) | 5% (n=1) | 11% (n=4) | 0.4250 | 27% (n=8) | 18% (n=4) | 0.4731 |
|  | Moderate or Severe Liver Disease | 0% (n=0) | 3% (n=1) | 0.4455 | 10% (n=3) | 9% (n=2) | 0.9125 |
|  | Metastatic Solid Tumor | 10% (n=2) | 6% (n=2) | 0.5560 | 10% (n=3) | 5% (n=1) | 0.4658 |
|  | AIDS/HIV | 0% (n=0) | 0% (n=0) | 1.0000 | 3% (n=1) | 0% (n=0) | 0.3872 |
|  | Charlson Comorbidity Score (median) | 4 | 5 | 0.8533 | 6 | 5 | 0.4286 |
| Encounter | Hospital LOS (median, days) | 46 | 36 | 0.3099 | 42 | 37 | 0.4431 |
|  | ICU during encounter | 100% (n=20) | 97% (n=34) | 0.4455 | 60% (n=18) | 45% (n=10) | 0.2986 |
|  | ICU at the time of CLABSI | 95% (n=19) | 86% (n=30) | 0.2880 | 30% (n=9) | 27% (n=6) | 0.8302 |
|  | Time to CLABSI (median, days) | 17 | 16 | 0.5752 | 20 | 18 | 0.4813 |
|  | In-house mortality | 40% (n=8) | 54% (n=19) | 0.3080 | 17% (n=5) | 27% (n=6) | 0.3549 |
| Line details | Central line duration during the encounter (median, days) | 24 | 22 | 0.3098 | 26 | 26 | 0.8312 |
|  | Device Utilization Ratio (catheter days/patient days) | 0.63 | 0.68 | **0.0148** | 0.58 | 0.55 | **0.0254** |
|  | Dialysis line attributed to CLABSI | 30% (n=6) | 37% (n=13) | 0.5920 | 43% (n=13) | 36% (n=8) | 0.6128 |
|  | Femoral line attributed to CLABSI | 10% (n=2) | 3% (n=1) | 0.2618 | 27% (n=8) | 18% (n=4) | 0.4731 |
|  | PICC line attributed to CLABSI | 25% (n=5) | 0% (n=0) | **0.0019** | 27% (n=8) | 27% (n=6) | 0.9612 |
|  | Port line attributed to CLABSI | 0% (n=0) | 3% (n=1) | 0.4455 | 17% (n=5) | 9% (n=2) | 0.4291 |
|  | PA catheter line attributed to CLABSI | 0% (n=0) | 0% (n=0) | 1.0000 | 7% (n=2) | 5% (n=1) | 0.7459 |
|  | Arterial line present on infection date | 90% (n=18) | 89% (n=31) | 0.8701 | 23% (n=7) | 32% (n=7) | 0.4956 |
| Organism | Gram-Positive organisms | 45% (n=9) | 57% (n=20) | 0.3856 | 47% (n=14) | 64% (n=14) | 0.2252 |
|  | Methicillin resistant *Staphylococcus aureus* | 5% (n=1) | 0% (n=0) | 0.1819 | 0% (n=0) | 5% (n=1) | 0.2383 |
|  | Methicillin susceptible *Staphylococcus aureus* | 0% (n=0) | 0% (n=0) | 1.0000 | 10% (n=3) | 5% (n=1) | 0.4658 |
|  | Coagulase-negative *Staphylococcus* | 15% (n=3) | 29% (n=10) | 0.2544 | 13% (n=4) | 36% (n=8) | 0.0515 |
|  | Enterococcus spp | 20% (n=4) | 29% (n=10) | 0.4827 | 17% (n=5) | 18% (n=4) | 0.8865 |
|  | Gram-Negative organisms | 10% (n=2) | 11% (n=4) | 0.8701 | 27% (n=8) | 27% (n=6) | 0.9612 |
|  | Candida spp | 50% (n=10) | 43% (n=15) | 0.6088 | 30% (n=9) | 14% (n=3) | 0.1665 |
|  | Polymicrobial CLABSI | 15% (n=3) | 17% (n=6) | 0.8363 | 3% (n=1) | 14% (n=3) | 0.1684 |
